# Combining *Wolbachia*-induced sterility and virus protection to fight *Aedes albopictus*-borne viruses

**DOI:** 10.1371/journal.pntd.0006626

**Published:** 2018-07-18

**Authors:** Riccardo Moretti, Pei-Shi Yen, Vincent Houé, Elena Lampazzi, Angiola Desiderio, Anna-Bella Failloux, Maurizio Calvitti

**Affiliations:** 1 Biotechnology and Agroindustry Division, ENEA (Italian National Agency for New Technologies, Energy and Sustainable Economic Development), Casaccia Research Center, Rome, Italy; 2 Department of Virology, Institut Pasteur, Arboviruses and Insect Vectors Unit, Paris, France; Oxford University Clinical Research Unit, VIETNAM

## Abstract

Among the strategies targeting vector control, the exploitation of the endosymbiont *Wolbachia* to produce sterile males and/or invasive females with reduced vector competence seems to be promising. A new *Aedes albopictus* transinfection (AR*w*P-M) was generated by introducing *w*Mel *Wolbachia* in the AR*w*P line which had been established previously by replacing *w*AlbA and *w*AlbB *Wolbachia* with the *w*Pip strain. Various infection and fitness parameters were studied by comparing AR*w*P-M, AR*w*P and wild-type (S_ANG_ population) *Ae*. albopictus sharing the same genetic background. Moreover, the vector competence of AR*w*P-M related to chikungunya, dengue and zika viruses was evaluated in comparison with AR*w*P. AR*w*P-M showed a 100% rate of maternal inheritance of *w*Mel and *w*Pip *Wolbachia*. Survival, female fecundity and egg fertility did not show to differ between the three *Ae*. *albopictus* lines. Crosses between AR*w*P-M males and S_ANG_ females were fully unfertile regardless of male age while egg hatch in reverse crosses increased from 0 to about 17% with S_ANG_ males aging from 3 to 17 days. When competing with S_ANG_ males for S_ANG_ females, AR*w*P-M males induced a level of sterility significantly higher than that expected for an equal mating competitiveness (mean Fried index of 1.71 instead of 1). The overall *Wolbachia* density in AR*w*P-M females was about 15 fold higher than in AR*w*P, mostly due to the *w*Mel infection. This feature corresponded to a strongly reduced vector competence for chikungunya and dengue viruses (in both cases, 5 and 0% rates of transmission at 14 and 21 days post infection) with respect to AR*w*P females. Results regarding Zika virus did not highlight significant differences between AR*w*P-M and AR*w*P. However, none of the tested AR*w*P-M females was capable at transmitting ZIKV. These findings are expected to promote the exploitation of *Wolbachia* to suppress the wild-type *Ae*. *albopictus* populations.

## Author summary

*Aedes albopictus* is one of the major human disease vectors and, despite substantial control efforts, it is rapidly spreading worldwide and increasing its epidemiological role. Thus, innovative approaches to fight this mosquito are urgently needed. Among the available control strategies, the exploitation of the endosymbiotic bacterium *Wolbachia* seems to be promising. In nature, the infection by *Wolbachia* is generally not detrimental, instead, it causes a series of modifications in host physiology promoting the spread of the infection in uninfected populations. Herein, we report on the artificial transinfection of specific *Wolbachia* strains in *Ae*. *albopictus* to replace its native *Wolbachia* infection type. This manipulation aimed at exploiting the expected modifications in the reproductive biology and vector competence of the species to contribute to reduce its epidemiological role. Specifically, we found that the new double *Wolbachia* infection did not affect *Ae*. *albopictus* fitness. The males belonging to the manipulated line, AR*w*P-M, induced full egg infertility in the wild-type females they mate with and showed increased male mating competitiveness. Remarkably, the AR*w*P-M females demonstrated significantly reduced competence for chikungunya and dengue viruses while both tested *Ae*. *albopictus* lines showed a very low susceptibility for Zika virus. These findings may encourage the use of AR*w*P-M *Ae*. *albopictus* as a highly efficient and safe biocide to suppress the wild-type populations.

## Introduction

Despite control measures applied worldwide over decades, arthropod-borne diseases continue to pose a constant threat to human and domestic animal health [1]. Human-induced changes in the environment, climate change, passive transportation and acquisition of resistance to insecticides by the vectors are contributing to a dramatic re-emergence of harmful viruses such as dengue (DENV) and yellow fever (YFV) (both *Flavivirus*, Flaviviridae) transmitted by mosquitoes [2,3,4]. As well, further pathogens are rapidly spreading in areas where suitable vectors and environmental conditions are present and are showing a day by day increasing status of pathogenic relevance. These are the cases of chikungunya (CHIKV; *Alphavirus*, Togaviridae) and Zika (ZIKV; *Flavivirus*, Flaviviridae) viruses [5,6,7].

*Aedes* spp. (Diptera: Culicidae) are considered the key vectors of DENV, YFV, CHIKV and ZIKV [8,9]. At present, *Ae*. *aegypti* seems to play a leading role as vector among all of the *Aedes* species, mainly due to its high anthropophily and preference for the urban areas of the tropical regions [10]. However, though generally considered a secondary vector when *Ae*. *aegypti* is present, *Ae*. *albopictus*, the Asian tiger mosquito, demonstrated a key epidemiological role when abundant [11,12]. Moreover, the species may be responsible of increased risks of epidemics in temperate climate areas [13], as demonstrated by the DENV [14,15] and CHIKV [16] outbreaks occurred in Europe in recent years. In fact, even if less adapted to survive in dry conditions compared to *Ae*. *aegypti*, *Ae*. *albopictus* eggs display a remarkable cold hardiness in the diapausing form [17] which is highly contributing to the impressive extension of the geographic distribution of the species [18]. In addition, a recent mutation in an envelope glycoprotein led to a significant increase in CHIKV infectivity for *Ae*. *albopictus* and enhanced dissemination in mosquito organs and transmission [19,20]. *Ae*. *albopictus* was also found susceptible to ZIKV [21,22,23] even if vector competence can be considered low [24]. At the time of writing, CHIKV outbreaks occurred in Lazio (Rome Province) and Calabria regions [25] are still recent, with nearly 300 confirmed cases [26], endorsing the urgency of renewed control approaches.

Besides insecticide spraying, various alternative mosquito control methods are being developed and experimented [27,28,29]. In particular, theoretical and experimental studies are showing that certain strategies targeting mosquito reproduction biology have the potential to significantly affect mosquito populations, leading to a diminished risk that they may support diseases [30,31]. Basically, these methods rely on the release of functionally sterile males produced by three main techniques, namely, the irradiation of pupae by γ- or x-rays [32,33], the introduction of lethal factors through genetic modification [34,35] and the manipulation of the insect microbiome by the transinfection of the symbiotic bacterium *Wolbachia* (Rickettsiales) [36]. A further control strategy once again involves *Wolbachia* and it is not based on the suppression of the vector population but instead on the gradual replacement of the wild-types with conspecifics displaying desired biological traits [37] as more thoroughly described below.

*Wolbachia* is a vertically transmitted endosymbiotic bacterium, quite common in arthropods and a few other invertebrate taxa [38], which mainly infects the germ line of both sexes and manipulates host reproduction promoting the spread of the infected individuals in uninfected populations [39]. Among the various *Wolbachia*-induced effects on host biology, Cytoplasmic Incompatibility (CI) occurs at early stages of embryonic development and characterizes unfertile crosses between individuals with different *Wolbachia* infection types [40]. Introducing artificially a CI-inducing strain of *Wolbachia* in a vector species may provide a tool to produce functionally sterile males to be used to compromise the fertility of wild-type females not infected by the above *Wolbachia* strain.

*Wolbachia*-based strategies for vector control started to encounter a significant record of success in recent years. This is mainly due to the property shown by certain *Wolbachia* strains to reduce the vector competence of newly infected mosquito species [41,42,43,44]. This principle has been applied with *Ae*. *aegypti*, which is not infected by *Wolbachia* in the wild, through the artificial introduction of a *Wolbachia* strain (*w*Mel) caught from *Drosophila melanogaster* (Diptera: Drosophilidae) [43]. This manipulation proved to suppress the DENV replication in the infected individuals and is responsible for a 70% reduction of the vector competence of this *Ae*. *aegypti* line [45]. A specific ongoing program aims at fighting dengue through the replacement of the wild-type *Ae*. *aegypti* population with this manipulated line [46]. The replacement is made feasible by the CI phenomenon which favors the *Wolbachia* infected over the uninfected *Ae*. *aegypti*. The *w*Mel infected *Ae*. *aegypti* also displayed reduced vector competence for ZIKV [47] and CHIKV [48].

*Ae*. *albopictus* is a competent vector for the above mentioned viruses despite being naturally infected with two *Wolbachia* strains (*w*AlbA and *w*AlbB). However, the introduction of the *w*Mel *Wolbachia* strain in a *Wolbachia*-cured line of *Aedes albopictus* induced resistance to DENV and CHIKV [49,50].

*w*Mel *Wolbachia* had been previously introduced in wild-type *Ae*. *albopictus*, obtaining a triple infection which showed detrimental effects on female fitness leading to the early loss of the transinfected line [51]. Shortly after, AR*w*P *Ae*. *albopictus* was produced through the introduction of *w*Pip *Wolbachia* belonging to the IV Incompatibility group [52] from *Culex pipiens* in a *Wolbachia-*cured population from Central Italy [53]. The obtained line showed a bidirectional incompatibility pattern with wild-type *Ae*. *albopictus* and was found highly efficient in suppressing this vector under laboratory [54,55] and semi-field settings [56]. Remarkably, compared to wild-type individuals belonging to the same genetic background, AR*w*P males displayed a significantly better male mating competitiveness under semi-field conditions in large enclosures [56]. Differently from *w*Mel, *w*Pip *Wolbachia* was proved to not significantly reduce *Ae*. *albopictus* capability to transmit CHIKV compared to wild-type females (Calvitti and Failloux, previously unpublished data, 2011; S1 Fig).

Herein, we report on the transinfection of *w*Mel *Wolbachia* in AR*w*P to combine the remarkable suitability to the mass rearing protocols and male mating competitiveness, shown by this *Ae*. *albopictus* line over more than 100 generations, with a reduction in the vector competence, as expected by the introduction of *w*Mel *Wolbachia*. This research aims to obtain an innovative and safe tool to suppress and/or replace *Ae*. *albopictus* wild-type populations based on considerations and conditions discussed below.

## Materials and methods

### Mosquito lines and rearing

Mosquito lines used in the experiments shared the same genetic background. S_ANG_ is a wild-type strain of *Ae*. *albopictus* colonized by using ovitraps in Anguillara Sabazia (Rome) in 2006 and since then reared under laboratory conditions at ENEA-Casaccia Research-Center (Rome). AR*w*P is a CI-inducing line, established at ENEA in 2008 through the transinfection of *Wolbachia*-cured S_ANG_ individuals with *w*Pip *Wolbachia* from *Culex pipiens* [53] and reared for about 100 generations under rearing settings described below. Both the lines described above were periodically outcrossed with wild-type individuals from the same area to preserve the genetic variability according to methods reported previously [55]. Specifically, virgin AR*w*P and S_ANG_ females were crossed every five generations with the same number of two weeks old males obtained from Anguillara wild-caught females. AR*w*P-M has been obtained through the transinfection of AR*w*P with *w*Mel *Wolbachia* from *D*. *melanogaster* as reported in a further paragraph.

Larvae were brought to adulthood inside 0.5 litre larval trays at the density of 1 larva/1 ml, augmented with a powder obtained by crushing dry cat food (Friskies Adults) at a fixed dose of 4 mg/larva of which 10% was given on day 1, 45% on day 2 and 45% on day 5. Adult mosquitoes were kept inside 40x40x40 cm cages at T = 28±1 C°, RH = 70±10%, L:D = 14:10 hours and were supplied with water and sucrose. Blood meals were provided through the use of anesthetized mice in agreement with the Bioethics Committee for Animal Experimentation in Biomedical Research and following procedures approved by the ENEA Bioethical Committee according to the EU directive 2010/63/EU. Used mice belonged to a colony housed at CR ENEA Casaccia and maintained for experimentation based on the authorization N. 80/2017-PR released on February the 2^nd^ 2017 by Italian Ministry of Health.

### *w*Mel *Wolbachia* transinfection in AR*w*P *Aedes albopictus* and vertical transmission

AR*w*P *Ae*. *albopictus* embryos were transinfected according to techniques already used for mosquito transinfection [53,57,58]. *D*. *melanogaster* belonging to the *yw*^*67C23*^ genotype [59] was kindly furnished by Luis Teixeira (Instituto Gulbenkian de Ciência, Oeiras, Portugal) to be used as *w*Mel *Wolbachia* donor. Cytoplasm was withdrawn from the posterior pole of donor eggs by borosilicate needles (Sutter Instrument; Novato, CA, USA) and then injected into the posterior of the recipient embryos using MN-151/MMO-202ND micromanipulators and an IM300 microinjector (Narishige Scientific; Tokyo, Japan).

After 5 days of development, the eggs were hatched by using a nutrient broth medium [60] and larvae were reared to the adult stage. G_0_ females, isolated as pupae to assure virginity, were mated with AR*w*P males and then provided with a blood meal. After oviposition, the infection status of G_0_ females and males was ascertained by PCR analysis using the *w*Mel-*wsp* loci primers [61]. In the case of a positive result, the obtained amplicons were sequenced to confirm the *Wolbachia* infection type. The progeny produced by infected females were selected to establish a new transinfected *Ae*. *albopictus* line, AR*w*P-M. To reduce the inbreeding effects, AR*w*P-M females were outcrossed with AR*w*P males for five generations. During the AR*w*P line establishment, the first 6 generations were monitored for transmission efficiency of *Wolbachia* infection. All the G_1_ adults were PCR assayed for presence of *w*Pip *Wolbachia* and infected offspring were chosen to start a new generation. Starting from G_2_, the maternal inheritance rate was estimated by assaying 5 daughters and 5 sons for each of three isolated females (mothers), randomly chosen.

### Fitness parameters

Adult survival, female fecundity and egg fertility of the AR*w*P-M line were measured in comparison with S_ANG_ and AR*w*P *Ae*. *albopictus*. Namely, each treatment consisted of 50:50 females:males in 40x40x40 cages furnished with 10% sugar solution and under climatic conditions reported above. Dead mosquitoes were counted and removed every four days to assess longevity until the test was stopped at 60 days.

At 1-week intervals and starting with 3±1 days-old females, a blood meal was provided and mosquito eggs were collected on wet germination paper until 7^th^ day after feeding. The eggs produced by the 3±1 days old females were counted and then hatched to measure female fecundity and mean egg fertility in the three lines. Each treatment was replicated three times.

### Cytoplasmic incompatibility and male mating competitiveness

Three different series of crossing experiments were set up to evaluate the CI pattern between AR*w*P-M and S_ANG_ and to measure the male mating competitiveness of the AR*w*P-M males in comparison with the S_ANG_ males in 100×50×50 cm cages. For this purpose, respectively, 2±1 and 3±1 days-old females and males were used: i) 20:20 S_ANG_ males:females were allowed to mate in control crosses; ii) CI crosses consisted of populations of 20:20 AR*w*P-M males:S_ANG_ females; iii) populations of 20:20 S_ANG_ males:AR*w*P-M females were used to measure CI in the reciprocal cross; iv) competition crosses involved 20:20:20 AR*w*P males:S_ANG_ males:S_ANG_ females respectively. After 24 h, males were retrieved and females were provided with a blood meal. On the day of oviposition, females were isolated into plastic tubes furnished with wet paper for individualized egg laying. Produced eggs were counted and then allowed to hatch to measure CI. In the case of no hatching egg, females were checked for the presence of spermatozoa to ascertain the occurrence of a mating and virgins were excluded from the counts. CI crosses (ii and iii) were also repeated with males aged 10±1 and 17±1 days to investigate age-dependant changes in the incompatibility level. The degree of CI was computed using the corrected index of cytoplasmic incompatibility (CI_corr_) and the Fried competitiveness index, as described previously [55].

### DNA purification and quantitative qPCR to evaluate *w*Mel and *w*Pip *Wolbachia* density

Ten male and ten female individuals belonging to the AR*w*P-M line were aged 5–10 days and then analyzed for *w*Pip and *w*Mel *Wolbachia* titer in comparison with the AR*w*P line.

Total DNA was extracted from whole body of individual mosquitoes, using the ZR Tissue & Insect DNA Kit MicroPrep (Zymo Research, Irvine, CA, USA), according to manufacturer instructions. Strain-specific primers were used to amplify the *w*Pip-*wsp* and *w*Mel-*wsp* loci (Zhou et al., 1998), using previously described oligonucleotides: *w*PF (CGACGTTAGTGGTGCAACATTTA) and *w*PR (AATAACGAGCACCAGCAAAGAGT) [54] to obtain a 272 bp fragment of the *w*Pip-*wsp* gene; 308F (TTA AAG ATG TAA CAT TTG) [61] and QArev2 (CAC CAG CTT TTA CTT GAC C) [62] leading to a 219 bp fragment of the *w*Mel-*wsp* gene.

*Aedes albopictus* actin gene was used as a nuclear reference and amplified with the primers pair actAlbqPCRsense (CCCACACAGTCCCCATCTAC) and actAlbqPCRantisense (CGAGTAGCCACGTTCAGTCA), leading to a 119 bp amplification product.

Amplification reaction was prepared using the FluoCycle II SYBR Master Mix (Euroclone, Milano, Italy) in 20 μl final volume. Each mosquito extract was analyzed in triplicate using 2 μl total DNA extract as a reaction template. PCR was performed on ABI Prism 7100 (Applied Biosystems, Foster City, CA, USA) thermal cycler, optimizing the elongation temperature for each primer pair. Hence, the following amplification programs were applied: 5 min at 95°C, followed by 40 cycles of 15 sec at 95°C and 1 min at 60°C/52°C/62°C, for primer pair *w*PF-*w*PR/308F-QArev2/ actAlbqPCRsense-actAlbqPCRantisense, respectively. The presence of specific amplification products was verified with dissociation curves.

A plasmid (named pBS-M-P-act) containing single copy of *w*Pip-*wsp*, *w*Mel-*wsp* and *actin* was constructed to obtain a quantitative reference in qPCR amplifications. To this aim, specific DNA sequences encoding for *w*Pip-*wsp*, *w*Mel-*wsp* and *actin*, were cloned from total DNA extracts. The *actin* fragment (119 bp) was obtained by PCR using field-caught *Ae*. *albopictus* total DNA as a template and the primers pair actAlbqPCRsense/actAlbqPCRantisense. A 404 bp fragment of *w*Pip-*wsp* locus was amplified using field-caught *Culex pipiens* total DNA extract as a template and the primers pair 183F/*w*PF [54,61], while a 405 bp fragment of *w*Mel-*wsp* locus was amplified using *D*. *melanogaster* total DNA extract and primers pair 308F/691R [61]. All amplicons were then cloned in pCR 2.1 (TA Cloning Kit, Invitrogen, Carlsbad, CA) plasmid vector.

The amplified sequences were then assembled in a single plasmid, according to the following procedure. *Actin* gene fragment was transferred from pCR 2.1 into BamHI-NotI sites of pBluescript II SK (+) vector, resulting in pBS-act plasmid. Then, *w*Pip-*wsp* fragment was cloned from pCR 2.1 into NotI-SacI sites of pBS-act, obtaining pBS-P-act plasmid. Finally, *w*Mel-*wsp* fragment was cloned from pCR 2.1 into KpnI-XhoI sites of pBS-P-act, resulting in pBS-M-P-act plasmid. All obtained constructs were sequenced to assess the correct assembling and the absence of unwanted sequence variations.

For qPCR quantitation the pBS-M-P-act plasmid was serially diluted to build a standard curve with all three loci present at an equimolar concentration. The same standard dilutions were used in each qPCR, in order to standardize the signal with the nuclear *actin* reference. Quantitative PCR amplification was performed in triplicate for each mosquito extract and mean genome number of *w*Pip-*wsp* and *w*Mel-*wsp* was obtained per nuclear *actin* copy number.

Accession numbers for the genes mentioned in the paragraph are reported in S1 Table.

### Vector competence tests for chikungunya, dengue and zika viruses

AR*w*P-M vector competence for CHIKV, DENV and ZIKV viruses was evaluated in comparison with AR*w*P *Ae*. *albopictus* to ascertain whether the introduction of the *w*Mel *Wolbachia* infection may affect this biological trait.

#### Viruses

CHIKV (CHIKV 06.21; accession number AM258992) was isolated in 2005 from a newborn male from La Reunion presenting meningo-encephalitis symptoms [63]. This strain belongs to the East-Central-South African (ECSA) lineage known to be better adapted to *Ae*. *albopictus* due to the E1-A226V mutation [19,20] and this genotype was involved in the 2007 outbreak in Emilia-Romagna Region (Italy) [64]. We assumed that the widespread of this CHIKV strain was a valid argument to chose it over others as more suitable to be involved in severe epidemics. DENV (DENV-1 1806; accession number EU482591) was obtained in 2010 from an autochthonous case in Nice, France [14]. ZIKV (ZIKV PE243; accession number KX197192) was isolated from a patient in Recife (Brazil) in 2015 [65]. Viral stocks were prepared after several passages of the isolate onto *Ae*. *albopictus* C6/36 cells for CHIKV and DENV, and Vero cells for ZIKV.

#### Experimental infections and viral titrations

One-week-old mosquitoes were isolated in boxes (60 females/box) and starved for 24 h before infection. The blood meal was composed of two parts of washed rabbit erythrocytes, one part of the viral suspension and a phagostimulant (ATP) at 5 mM. The infectious blood-meal at a viral titer of 10^7^FFU/mL for CHIKV and DENV-1 and, 10^7^PFU/mL for ZIKV was placed in capsules (Hemotek, Lancashire, UK) wrapped with a piece of pork intestine maintained at 37°C. After 15–20 min of feeding, engorged females were sorted on ice and incubated at 28°C, 80% RH and 16h:8h L:D cycle, with free access to 10% sucrose. Batches of 20–24 mosquitoes were examined at 7 and 14 days post-infection (dpi) for CHIKV, and 14 and 21 dpi for DENV-1 and ZIKV. Mosquitoes were processed as follows: abdomen and thorax (referred to as body) were examined to determine infection, head for dissemination and saliva for transmission. Infection rate (IR) corresponds to the proportion of mosquitoes with infected midgut, dissemination efficiency (DE) to the percentage of mosquitoes with virus detected in heads suggesting a successful viral dissemination from the midgut, and transmission efficiency (TE) to the proportion of mosquitoes with infectious saliva. IR, DE and TE were calculated by titrating body, head homogenates, and saliva, respectively.

To determine viral infection and dissemination rates, each mosquito body and head were ground in 300 μL of medium (Leibovitz L15 medium for CHIKV and DENV, and Dulbecco’s Modified Eagle medium (DMEM) for ZIKV) supplemented with 2% fetal bovine serum (FBS), centrifuged at 10,000 × g for 5 min at +4°C and inoculated onto monolayers of *Ae*. *albopictus* C6/36 cell culture (for CHIKV and DENV) or Vero cells (for ZIKV) in 96-well plates. Vero cells were incubated for 7 days at 37°C then stained with a solution of crystal violet (0.2% in 10% formaldehyde and 20% ethanol). Presence of viral particles was assessed by detection of CPE. C6/36 cells were incubated for 3 days (CHIKV) or 5 days (DENV) at 28°C and then were fixed with 10% formaldehyde, washed, and revealed using hyper-immune ascetic fluid as the primary antibody and Alexa Fluor 488 goat anti-mouse IgG as the second antibody (Life Technologies).

To estimate viral transmission, mosquito saliva was collected in individual pipette tips containing 5 μL FBS for 30 min as previously described [66]. FBS containing mosquito saliva was expelled into 45 μL of L15 medium, inoculated on C6/36 cell culture or Vero cells stained as described above.

#### Cell cultures

C6/36 (*Ae*. *albopictus*) cells used for CHIKV and DENV titrations were maintained at 28°C in L-15 medium supplemented with non-essential amino-acids (1X), 10% fetal bovine serum (FBS), 100 units/mL penicillin and 100 μg/mL streptomycin. Vero (green monkey kidney, ATCC CCL-81) cells used for ZIKV titrations were maintained at 37°C, 5% CO2 in DMEM with 10% FBS, 100 units/mL penicillin and 100 μ g/mL streptomycin.

### Data analysis

Survival curves of the three different lines (AR*w*P, S_ANG_, and AR*w*P-M) were compared using Kaplan-Meier method and log-rank (Mantel-Cox) test. One-way repeated-measures ANOVA and Bonferroni mean separation were used to compare fecundity and egg hatch data between lines. Percent data was transformed to arcsin square root of proportions before the analysis. Normality of the experimental data was determined by the Shapiro–Wilk test. ANOVA was also used to compare the mean level of observed and expected CI in the male competitiveness trials.

Difference between lines in infection rate (IR) dissemination efficiency (DE) and transmission efficiency (TE) were analyzed by the Fisher’s exact test while Kruskal-Wallis test was used to compare the mean number of viral particles detected in bodies and saliva.

Statistical analysis was performed by PASW statistics (PASW Statistics for Windows, Version 18.0. SPSS Inc., Chicago, USA).

## Results

### Transinfection results and vertical transmission

More than 900 *Ae*. *albopictus* embryos were microinjected in total and 12 eggs were viable after the treatment and gave first instar larvae. Among obtained larvae, 8 emerged as adults, 4 of which were found infected with *w*Mel *Wolbachia*. Two infected females were used to establish transinfected isofemale lines and one out of them transmitted the *w*Mel infection to the progeny. All of the tested G_1_ individuals were confirmed as positive for *w*Mel *Wolbachia* and vertical transmission accuracy always approached 100% over the following generations with few exceptions only among male progeny (98.89±1.01% in mean) (Table 1). The obtained line was named AR*w*P-M. The confirmation of the transinfected *Wolbachia* strain was achieved by sequencing the *wsp* gene [61] to perform a comparison with published *w*Mel *wsp* sequence (Accession Number: AF020064.1; S2 Fig).

**Table 1:** Maternal inheritance efficiency of the *w*Mel infection in the AR*w*P-M *Ae*. *albopictus* line. The data sheet shows the number (N) of analyzed and the percentage of infected male and female individuals at each generation following the *w*Mel transinfection.

		G_1_	G_2_	G_3_	G_4_	G_5_	G_6_	mean	SE
males	N	4	15	15	15	15	15		
	% infected	100	100	93.33	100	100	100	98.89	1.01
females	N	5	15	15	15	15	15		
	% infected	100	100	100	100	100	100	100	0

### Fitness parameters

**Fig 1:**
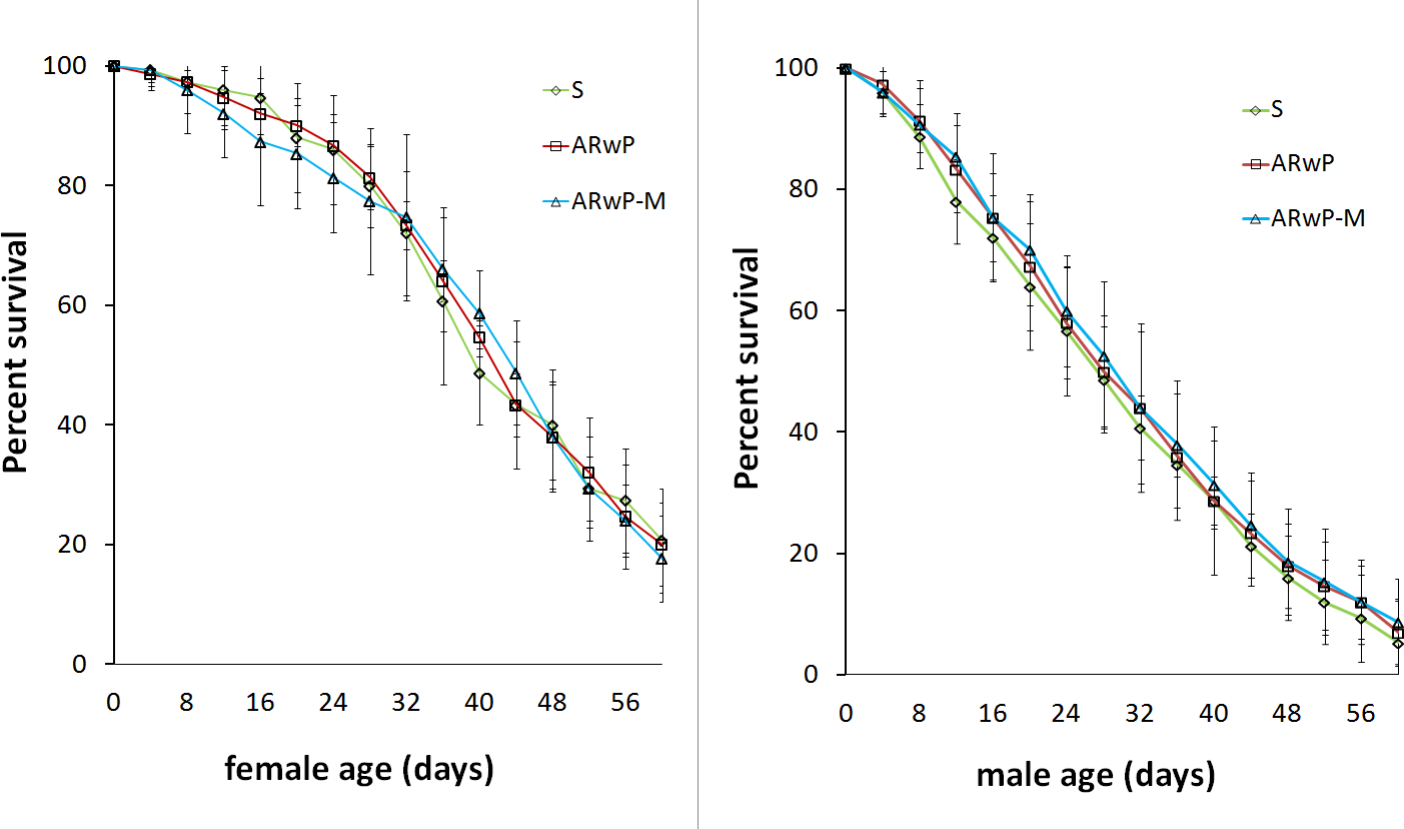
**Survival of AR*w*P-M females (left) and males (right) in comparison with recipient AR*w*P and wild-type *Ae*. *albopictus*.** S = S_ANG_ wild-type *Ae*. *albopictus*; AR*w*P = *w*Pip infected *Ae*. *albopictus*; AR*w*P-M *w*Pip + *w*Mel infected *Ae*. *albopictus*. Error bars show the SEM of three biological replicates, each containing 50:50 females:males. In both cases, survival curves did not show to significantly differ by Kaplan-Meier method and log-rank (Mantel-Cox) test.

Regardless of the sex, survival did not show to significantly differ between S_ANG_, AR*w*P and AR*w*P-M *Ae albopictus* (Fig 1). The average female life span was slightly higher than 38 days in all of the three *Ae*. *albopictus* lines under these experimental conditions (P = 0.984, log rank test). Average life expectancy for males was reduced to about 30 days in all of the tested lines (P = 0.984, log rank test).

**Fig 2:**
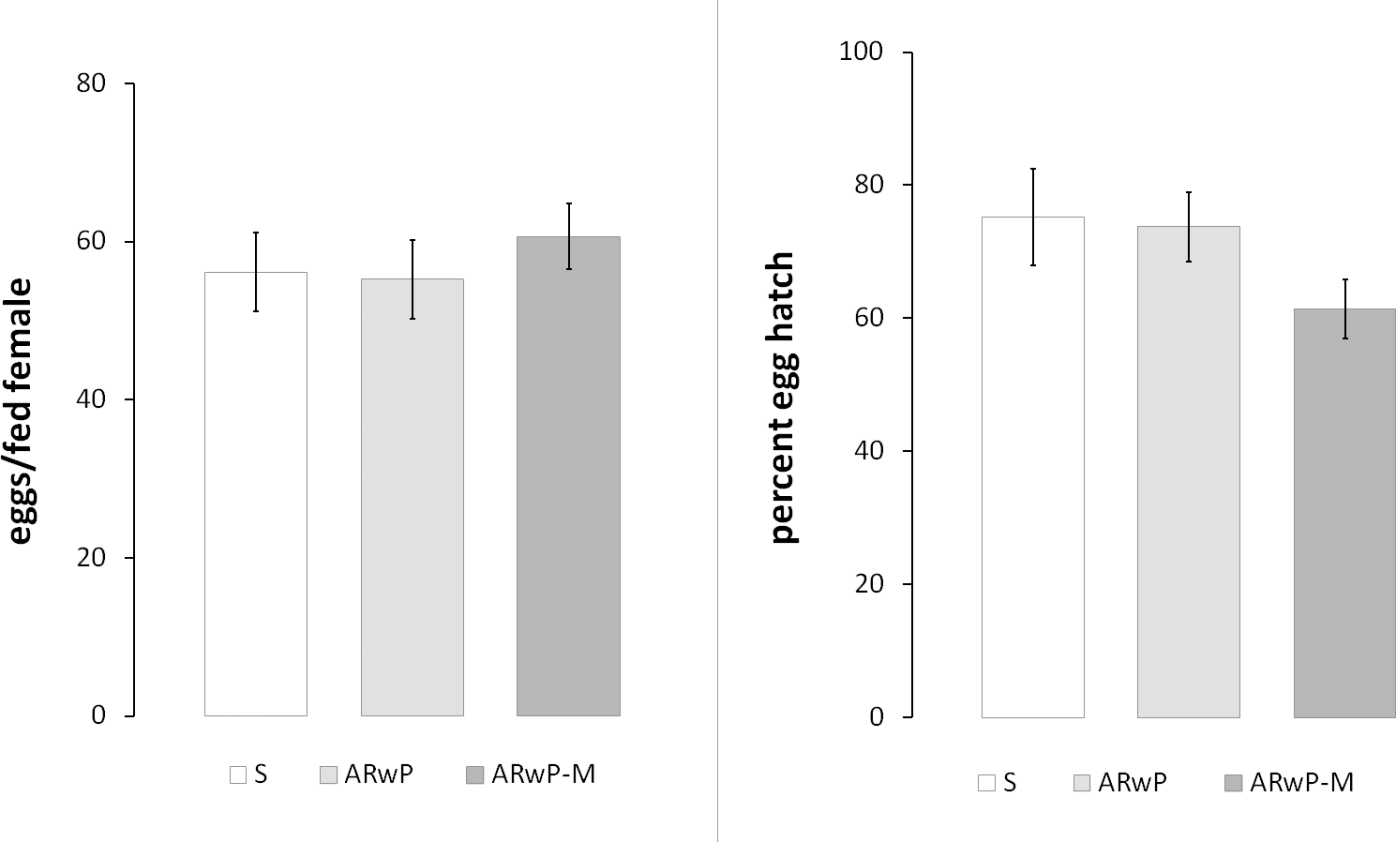
**Female fecundity (left) and hatch rate (right) in AR*w*P-M *Ae*. *albopictus* in comparison with recipient AR*w*P and wild-type *Ae*. *albopictus*.** S = S_ANG_ wild-type *Ae*. *albopictus*; AR*w*P = *w*Pip infected *Ae*. *albopictus*; AR*w*P-M *w*Pip + *w*Mel infected *Ae*. *albopictus*. Error bars show the SEM of three biological replicates, each containing 17–20 fed females. In both cases, values are not significantly different by ANOVA-Bonferroni (P > 0.05).

At AR*w*P-M G_8_, mean female fecundity did not significantly differ between tested *Ae*. *albopictus* lines (F_(2,6)_ = 0.005; P = 0.995; Fig 2). As well, the *w*Mel infection did not significantly affect AR*w*P-M fertility compared to both S_ANG_ and AR*w*P lines (F_(2,6)_ = 1.395; P = 0.318).

### CI and male mating competitiveness

Regardless of age, AR*w*P-M males compromised the hatchability of all of the eggs produced by the wild-type females they mated with (Table 2). Instead, the reverse crosses (S_ANG_ males × AR*w*P-M females) gave age-dependant results with egg fertility values gradually increasing from 0.09 ± 0.05 to 17.29 ± 2.32 when S_ANG_ males were, respectively, 3 and 17days (±1) old. AR*w*P-M males demonstrated higher mating competitiveness compared to the wild-types presenting the same genetic background as shown by the measured level of CI_corr_ and by the Fried competitiveness index (Table 2) which was significantly higher than 1 (F_(1,4)_ = 11.24; P = 0.028).

**Table 2:** Crosses between AR*w*P-M and wild-type *Ae*. *albopictus* (S_ANG_) to measure the level of induced cytoplasmic incompatibility and compare the male mating competitiveness. In all of the crosses, females were 2±1 days old. The CI_corr_ level in the CI crosses was measured at three different male ages. Competition crosses consisted of young (3 ±1 days old) AR*w*P-M and S_ANG_ males at 1:1 ratio.

crosses		N	percent egg hatch	CI_corr_	Fried index
females	males (*)				
S_ANG_	S_ANG_ (3)	2076	72.19 ± 3.12	0	
S_ANG_	AR*w*P-M (3)	2152	0.00 ± 0.00	100	
S_ANG_	AR*w*P-M (10)	2010	0.00 ± 0.00	100	
S_ANG_	AR*w*P-M (17)	1962	0.00 ± 0.00	100	
AR*w*P-M	S_ANG_ (3)	2175	0.09 ± 0.05	99.87 ± 0.07	
AR*w*P-M	S_ANG_ (10)	1982	12.84 ± 1.50	82.22 ± 2.08	
AR*w*P-M	S_ANG_ (17)	1985	17.29 ± 2.32	76.06 ± 3.21	
S_ANG_	1:1 S_ANG_:AR*w*P-M	2253	26.32 ± 2.25	62.07 ± 3.60	1.71 ± 0.24**

*in brackets, male ages (days±1) are specified

N = total number of screened eggs; mean percent egg hatch and SE represent three biological replicates; CI_corr_ calculation derives from the equation: CI_corr_(%) = [(CI_obs_ − CCM)/(100 − CCM)] × 100, where CCM represents the natural egg mortality in S_ANG_ control; the Fried index of male competitiveness is obtained from the equation: (N/S)[(H_n_-H_o_)/(H_o_-H_s_)] where N/S stands for the ratio between the males belonging to the two lines (in this case 1), H_n_ the egg hatch in compatible crosses, H_o_ the egg hatch in competition trials and H_s_ the egg hatch in the CI crosses.

**The Fried index of competitiveness is significantly higher than that expected for an equal competitiveness between S_ANG_ and AR*w*P-M males (P < 0.05, ANOVA).

### *Wolbachia* density in AR*w*P-M

**Fig 3:**
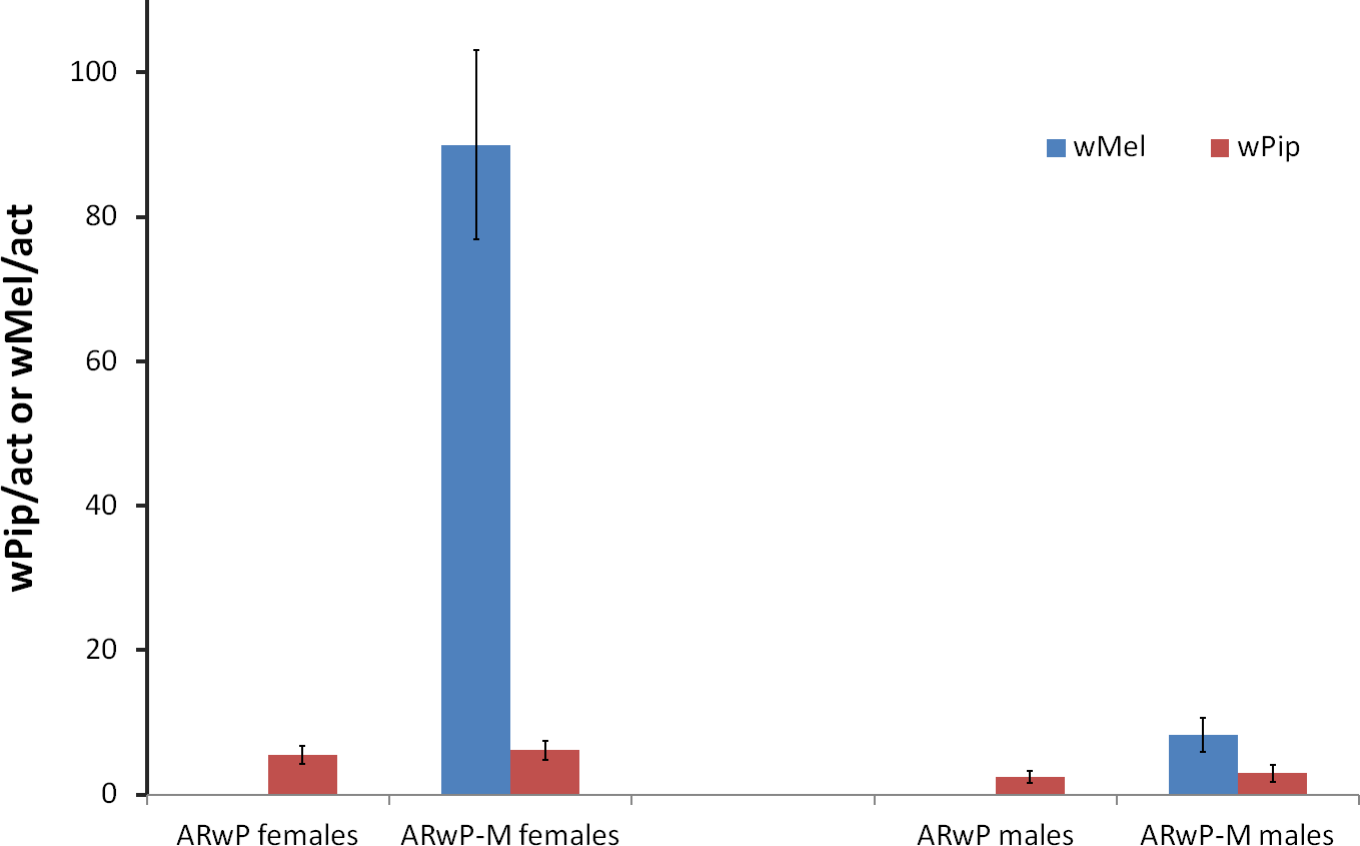
*w*Mel and *w*Pip *Wolbachia* density in AR*w*P and AR*w*P-M females and males measured by using *Ae*. *albopictus* actin gene as reference.

Adding *w*Mel *Wolbachia* to the AR*w*P line (*w*Pip-only infected) led to a significant increase in the overall *Wolbachia* titer (F_(1,18)_ = 51.346; P < 0.005) which, specifically, was about 15 fold higher in the AR*w*P-M compared to the AR*w*P females (Fig 3). The increase in *Wolbachia* density in AR*w*P-M males was less evident but significant as well (F_(1,18)_ = 12.673; P < 0.005). The titer of *w*Pip *Wolbachia* seemed to be not affected by the introduction of the additional *Wolbachia* strain (females: F_(1,18)_ = 0.133; P = 0.720; males: F_(1,18)_ = 0.136; P = 0.716).

### Vector competence

We experimentally infected mosquitoes with the three viruses, CHIKV, DENV and ZIKV provided to mosquitoes at a titer of 10^7^ FFU(PFU)/mL.

**Fig 4:**
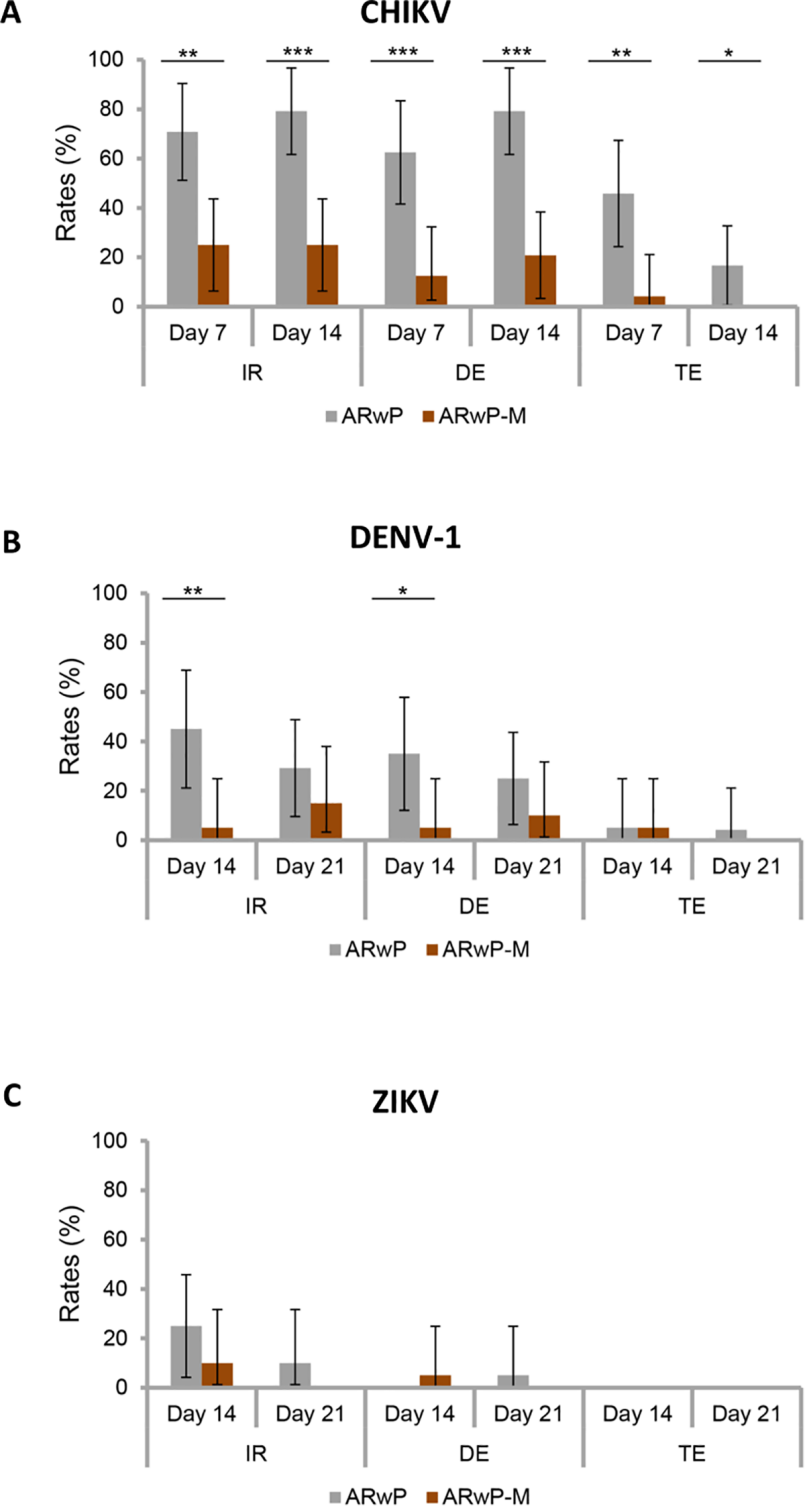
Rates of infection, dissemination efficiency and transmission efficiency for CHIKV, DENV and ZIKV in AR*w*P and AR*w*P-M *Ae*. *Albopictus*. IR = Infection rate; DE = Dissemination rate; TE = transmission rate; A: the differences between *Ae*. *albopictus* lines are significant with respect to all of the three parameters and at both time intervals (7, 14 dpi) post the infection (Fisher exact test, P < 0.05); B: AR*w*P and AR*w*P-M significantly differed with regard to IR and DE at 14 dpi (Fisher exact test, P < 0.05); C: AR*w*P and AR*w*P-M did not significantly differ with regard to any of the evaluated parameters.

When analyzing mosquitoes infected with CHIKV, significant differences were detected between the two *Ae*. *albopictus* lines at each dpi (7, 14) and parameters examined (IR, DE, TE) (Fig 4A). The AR*w*P line showed higher rates of infection, dissemination and transmission suggesting that AR*w*P was more susceptible to CHIKV than AR*w*P-M (Fisher exact test: P < 0.05). Previous results had shown that the vector competence for CHIKV was not significantly different comparing AR*w*P to S_ANG_ *Ae*. *albopictus* (Calvitti and Failloux, previously unpublished data, 2011; S1 Fig). Thus we can reasonably conclude that adding *w*Mel to AR*w*P led to a reduced vector competence for CHIKV also compared to the wild-types. In fact, about 5 and 0% of the infected AR*w*P-M females were able to transmit the virus, respectively, at 14 and 21 dpi.

When examining mosquitoes infected with DENV-1, only IR and DE at 14 dpi were significantly different between the two mosquito lines (Fig 4B). Again, AR*w*P was better infected and better disseminated by DENV-1 at 14 dpi compared to AR*w*P-M (Fisher exact test: P < 0.05).

When comparing mosquitoes infected with ZIKV, no significant differences were detected between the two *Ae*. *albopictus* lines (Fig 4C) with very low rates at 14 and 21 dpi.

**Fig 5:**
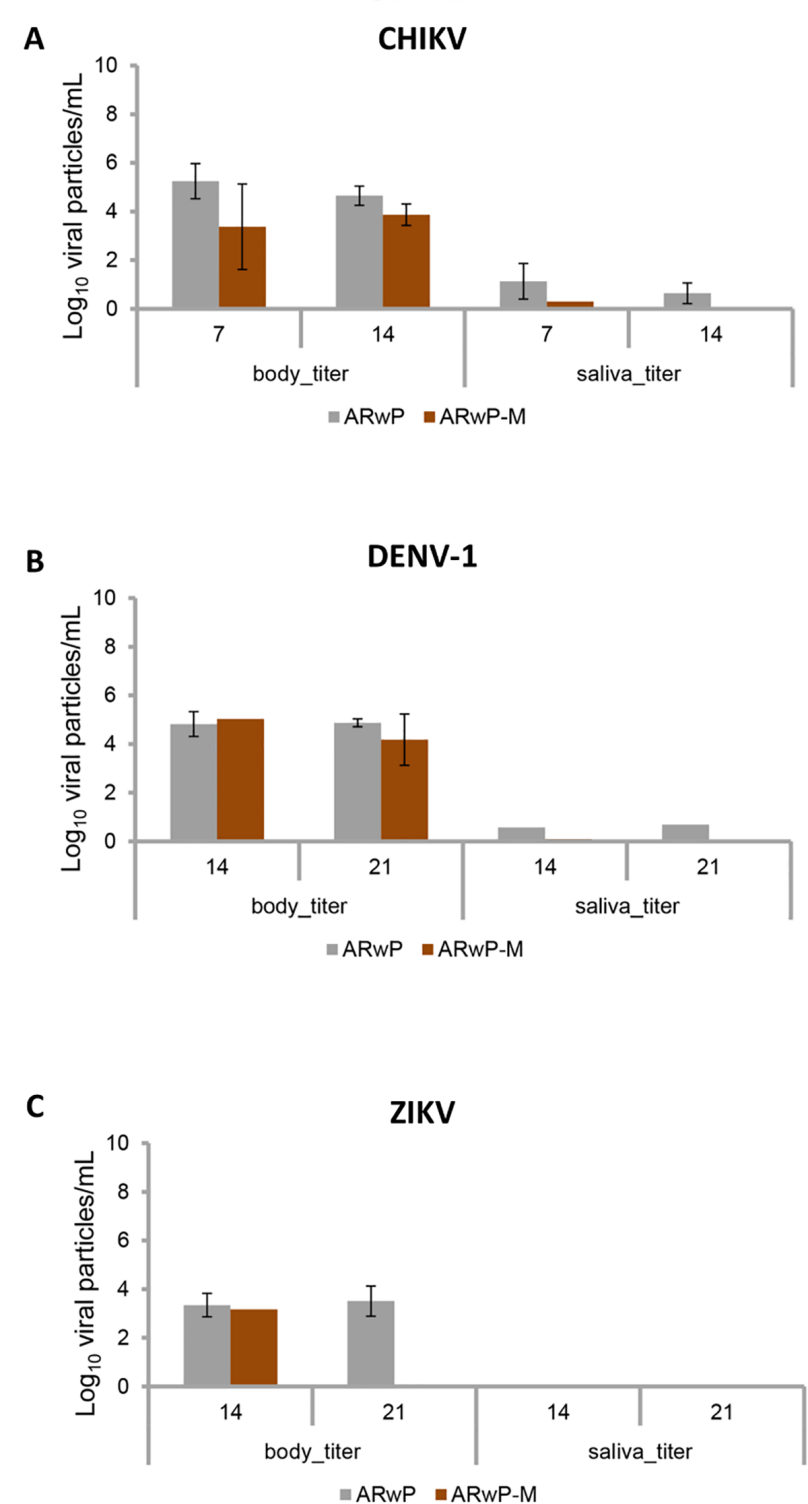
Titration of the viral particles of CHIKV, DENV and ZIKV in body and saliva of AR*w*P and AR*w*P-M *Ae*. *Albopictus*. The number of viral particles in the body and saliva of both mosquito lines were titrated for evaluating the viral load in each mosquito line. A: the number of CHIKV viral particles in the body and saliva of AR*w*P and AR*w*P-M at 7 and 14 dpi; B and C: the number of DENV-1 (B) and ZIKV (C) viral particles in the body and saliva of AR*w*P and AR*w*P-M at 14 and 21 dpi. Differences between *Ae*. *albopictus* lines were not statistically significant (Kruskal–Wallis test: P > 0.05).

When examining the number of viral particles detected in bodies and saliva (Fig 5A–5C), no significant differences were found between AR*w*P and AR*w*P-M (Kruskal–Wallis test: P > 0.05). Regarding CHIKV, very low values of viral particles were found in AR*w*P-M saliva at 14 dpi and this value decreased to 0 at 21 dpi. Regarding DENV and ZIKV, viral particles were undetectable in the saliva of AR*w*P-M females at both dpi.

## Discussion

The *Wolbachia*-based Incompatible Insect Technique (IIT) may offer a highly efficient approach to suppress mosquito vector populations because it can combine high efficacy with sustainable costs and negligible side-effects [67,68,69]. The efficiency of the approach has started to be demonstrated in the field with *Ae*. *albopictus* [70]. In this context, the introduction of different *Wolbachia* strains in a species may provide new resources among which to select the most suitable phenotypic effects for mosquito control purposes [71,72,73]. By introducing *w*Mel *Wolbachia* in AR*w*P, we hoped to retain certain useful traits characterizing the line while adding further beneficial biological features to increase its potential as a control tool of *Ae*. *albopictus*-borne diseases. Based on the obtained results, these expectations were fulfilled.

As already reported for a *w*Mel-only infected *Ae*. *albopictus* [49], the *w*Mel infection was not found to affect *Ae*. *albopictus* fitness even in the case of coexistence with *w*Pip *Wolbachia*. In addition, the CI trials demonstrated that AR*w*P-M maintained the notable male mating competitiveness already reported for AR*w*P in large enclosures under field conditions [56]. This advantage over the wild-types seemed to increase when moving from small cages to larger environments thus, we previously hypothesized that it could be due to AR*w*P male size [56,74]. However, this idea should be confirmed by more specific tests with regard to both AR*w*P and AR*w*P-M because changing environment could significantly affect the outcome of the trials. In any case, it is clear that releasing males with higher mating competitiveness compared to the wild-types may lead to induced infertility levels not reachable when using the same amount of irradiated males or males carrying dominant lethal mutations. This is because, using irradiation to obtain fully infertile *Ae*. *albopictus* males means reducing their mating competitiveness and survivorship, while preserving these latter traits by lowering the irradiation doses leads to a residual fertility which was found to increase with age [75]. Similarly, RIDL *Ae*. *aegypti* males showed reduced survivorship and mating competitiveness compared to the wild-types [76]. Furthermore, AR*w*P and, as confirmed herein, AR*w*P-M can be easily outcrossed, thanks to the partial fertility between the old wild-type males and the females belonging to these *Ae*. *albopictus* lines [77], allowing the preservation of the genetic variability and the transfer of the *w*Pip *Wolbachia* infection into local *Ae*. *albopictus* genotypes by introgression. This possibility may consent AR*w*P/AR*w*P-M to be adapted to local environmental conditions and to acquire useful mutations from the wild-types of the target areas such as the ones responsible for the insecticide-resistance [29].

Compared to SIT, exploiting *Wolbachia* to produce functionally sterile males could also save costs (radiation sources would be not needed) and reduce logistic problems (it would not be necessary to manipulate and transport mosquito pupae as needed by the sterilization procedure).

Aside from these obvious advantages attributable to IIT, the opportunity to set up application protocols based on male-only releases or not is highly debated [77,78,79]. In fact, since 100% efficient sexing methods are not yet available for *Aedes* mosquitoes, applying the IIT would mean releasing in the wild fertile females harboring a new *Wolbachia* infection type. Due to bidirectional CI and immigration, small AR*w*P/AR*w*P-M populations are not expected to establish and invade much larger wild-type *Ae*. *albopictus* populations as the eventual replacement would not be self-sustaining [56,77,79]. However, it is certain that it would be preferable to avoid releasing vector females in areas subjected to epidemics.

As expected, the introduction of *w*Mel in AR*w*P *Ae*. *albopictus* had a profound impact on the vector competence of this line. *w*AlbA and *w*AlbB *Wolbachia* were proved to not interfere with the transmission of CHIKV [80]. Also, we demonstrated previously (S1 Fig) that *w*Pip *Wolbachia* was not capable of blocking this virus. Instead, when introducing *w*Mel in AR*w*P *Ae*. *albopictus*, a blockade of CHIKV was detected lowering the potential of this mosquito to transmit the virus. This phenotype was shared with *Wolbachia*-cured *Ae*. *albopictus* transfected with *w*Mel [49].

Mounting experimental evidence suggests that the low vector competence of wild-type *Ae*. *albopictus* for DENV is correlated with the presence of the natural of *w*AlbA and *w*AlbB *Wolbachia* strains [81,82]. While removing these strains canceled the inhibition exerted by *Wolbachia* on DENV [82], we demonstrated that adding *w*Mel to *w*Pip imposed a higher reduction of DENV-1 transmission by AR*w*P-M *Ae*. *albopictus* compared to AR*w*P. An even higher level of refractoriness to DENV transmission was previously obtained in a *w*Mel-only infected *Ae*. *albopictus* [50], possibly due to a higher *w*Mel *Wolbachia* titer compared to AR*w*P-M. However, the *Wolbachia* density data reported in the latter article is only expressed as a ratio compared to the wild-types thus, a direct comparison with the results reported herein is not feasible.

Lastly, the effect of exogenous *Wolbachia* strains in *Ae*. *albopictus* susceptibility to ZIKV is difficult to apprehend as the basic level of *Ae*. *albopictus* competence for ZIKV is already very low compared to CHIKV and DENV [83,84,85]. Our results confirmed the above results also in the *Ae*. *albopictus* lines infected with *w*Pip alone or with *w*Pip and *w*Mel *Wolbachia*. However, the inhibition of ZIKV transmission seems to be significantly enhanced in both AR*w*P and AR*w*P-M *Ae*. *albopictus* compared to the wild-type *Ae*. *albopictus* from the same geographic area [83]. In fact, the above authors reported on ZIKV transmission rates of 29% in *Ae*. *albopictus* from Rome while, in this work, none of the tested AR*w*P-M females was capable of transmitting the virus.

Making available an *Ae*. *albopictus* line which couples high male mating competitiveness and suitability to the mass rearing protocols to a reduced vectorial competence would diminish the concerns associated with the possible escape of females among the released males in IIT programs. However, a series of issues will certainly need to be addressed before moving with AR*w*P-M to field testing. Further studies will have to evaluate AR*w*P-M vector competence in comparison with local wild-type populations and also testing other DENV and CHIKV serotypes. Moreover, the long-term stability of the new *Wolbachia* infection will be investigated because natural selection might gradually lead to reduced symbiont density and the loss of antiviral protection [86]. In particular, the suitability of the line to the stressing mass production protocols will be studied together with its response to the environmental conditions of the open field. In fact, *w*Mel *Wolbachia* is known to be quite susceptible to heat stress as *Ae*. *aegypti* eggs and larvae maintained at temperatures higher than 30°C showed a dramatic reduction of the *Wolbachia* titer [87,88]. Such temperatures are common at low latitudes as well as during the summer in the Mediterranean basin and they might lead to a reduced pathogen inhibition and to a progressive diminution or even loss of the infection.

## Supporting information

S1 Fig Transmission rate and CHIKV virus titer in saliva at 7 and 14 dpi in AR*w*P, S_ANG_ wild-type and *Wolbachia*-cured *Ae*. *albopictus*. *w*Pip = AR*w*P *Ae*. *albopictus*; *w*AlbA & *w*AlbB = S_ANG_ wild-type *Ae*. *albopictus*; w- = *Wolbachia*-cured S_ANG_; dpi = days post infection. Mosquitoes were infected with CHIKV at a titer of 10^7^ FFU(PFU)/mL. (A) Transmission rate was not significantly different between *Ae*. *albopictus* lines both at 7 and 14 dpi (Fisher exact test, P < 0.05). (B) Virus titer in female *Ae*. *albopictus* did not differ between lines at both 7 and 14 dpi (Kruskal–Wallis test: P < 0.05).
(PDF)

S2 Fig Sequence of the *wsp* locus of *w*Mel *Wolbachia* present in AR*w*P-M *Ae*. *albopictus*. The *wsp* gene was initially amplified by PCR, using *wsp* generic primers 81F and 691R [61]. The obtained amplicon (AR*w*P Mel-amplicon) was then sequenced using the 308F and QArev2 primers specific for *w*Mel. The grey box indicates the regions of sequence homology. *wsp* sequence of *w*Pip *Wolbachia* (*wsp w*Pip AF301010) was also reported to highlight sequence differences with the *w*Mel *wsp* locus (*wsp w*Mel AF020064.1). The perfect alignment of the obtained amplicon with the *w*Mel *wsp* gene demonstrated the presence of *w*Mel *Wolbachia* in the transinfected AR*w*P-M *Ae*. *albopictus* line.
(PDF)

S1 Table Assembling of plasmid pBS-M-P-act was performed by cloning fragments of the sequences of interest using field-caught insects total DNA extracts as PCR templates. The sequence analysis of the cloned sequences revealed a complete homology with the corresponding genes in database.
(PDF)
