# Supplementary figures and images for: Combining Wolbachia-induced sterility and virus protection to fight Aedes albopictus-borne viruses
Source: PLoS Negl Trop Dis. 2018 Jul 18;12(7):e0006626. doi: 10.1371/journal.pntd.0006626 (PMC6066253; doi:10.1371/journal.pntd.0006626)

*Ae. albopictus* at day 7 post-infection with CHIKV

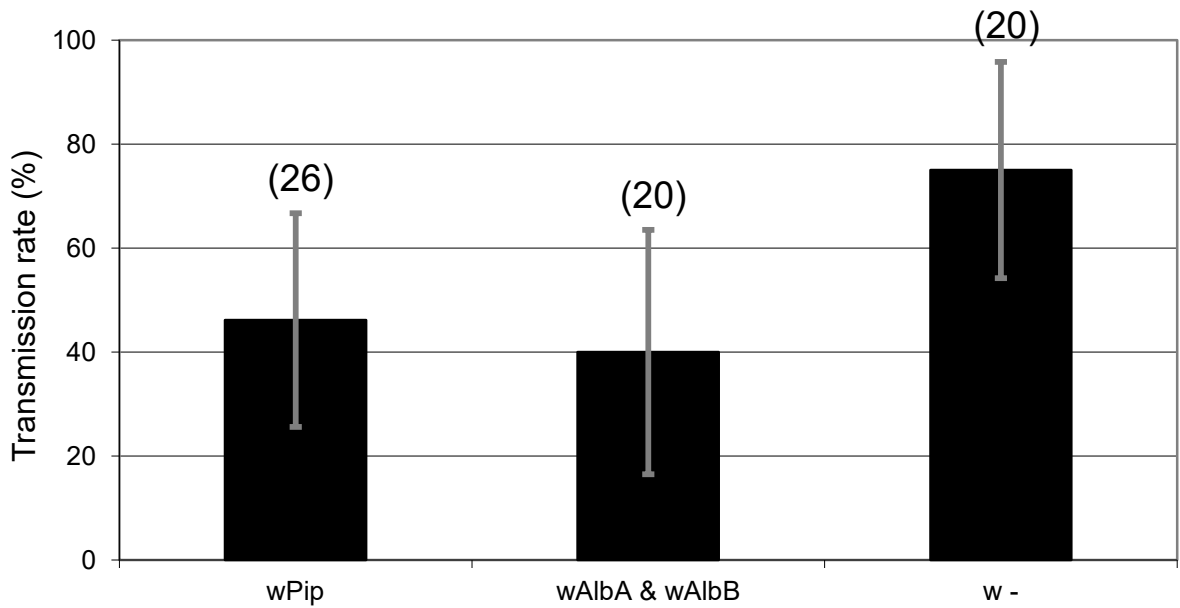

*Ae. albopictus* at day 14 post-infection with CHIKV

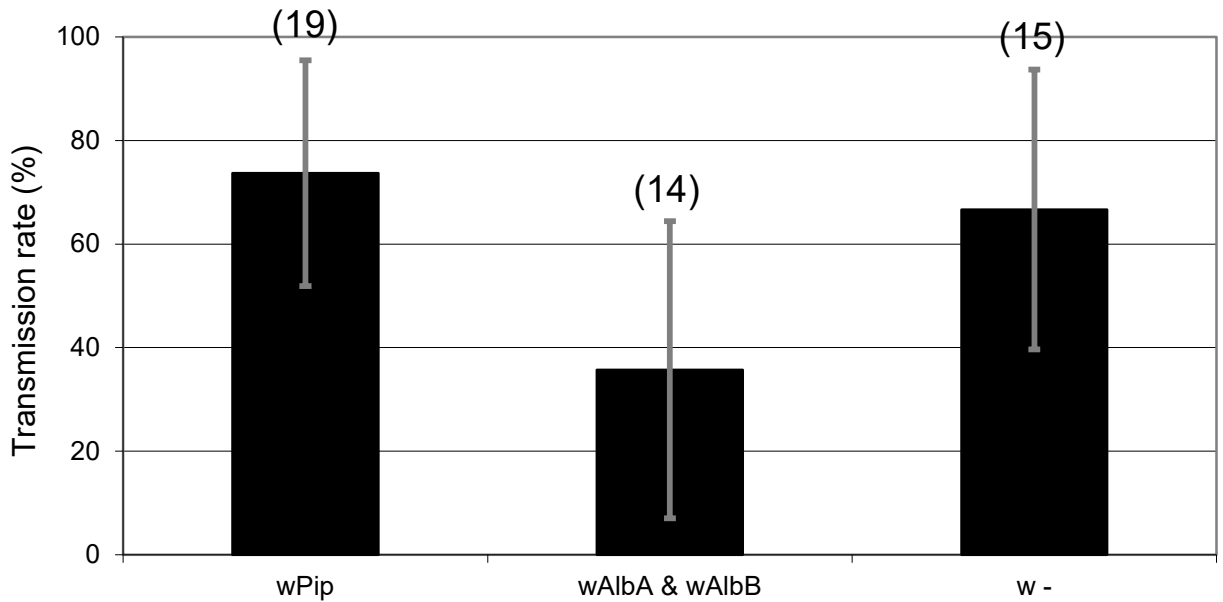

Supplement: S1 Fig — wPip = ARwP Ae. albopictus; wAlbA & wAlbB = SANG wild-type Ae. albopictus; w- = Wolbachia-cured SANG; dpi = days post infection. Mosquitoes were infected with CHIKV at a titer of 107 FFU(PFU)/mL. (A) Transmission rate was not significantly different between Ae. albopictus lines both at 7 and 14 dpi (Fisher exact test, P < 0.05). (B) Virus titer in female Ae. albopictus did not differ between lines at both 7 and 14 dpi (Kruskal–Wallis test: P < 0.05). (PDF) [file pntd.0006626.s001.pdf]
