# Supplementary material for: Combining Wolbachia-induced sterility and virus protection to fight Aedes albopictus-borne viruses
Source: PLoS Negl Trop Dis. 2018 Jul 18;12(7):e0006626. doi: 10.1371/journal.pntd.0006626 (PMC6066253; doi:10.1371/journal.pntd.0006626)
Supplement: S2 Fig — The wsp gene was initially amplified by PCR, using wsp generic primers 81F and 691R [61]. The obtained amplicon (ARwP Mel-amplicon) was then sequenced using the 308F and QArev2 primers specific for wMel. The grey box indicates the regions of sequence homology. wsp sequence of wPip Wolbachia (wsp wPip AF301010) was also reported to highlight sequence differences with the wMel wsp locus (wsp wMel AF020064.1). The perfect alignment of the obtained amplicon with the wMel wsp gene demonstrated the presence of wMel Wolbachia in the transinfected ARwP-M Ae. albopictus line. (PDF) [file pntd.0006626.s002.pdf]

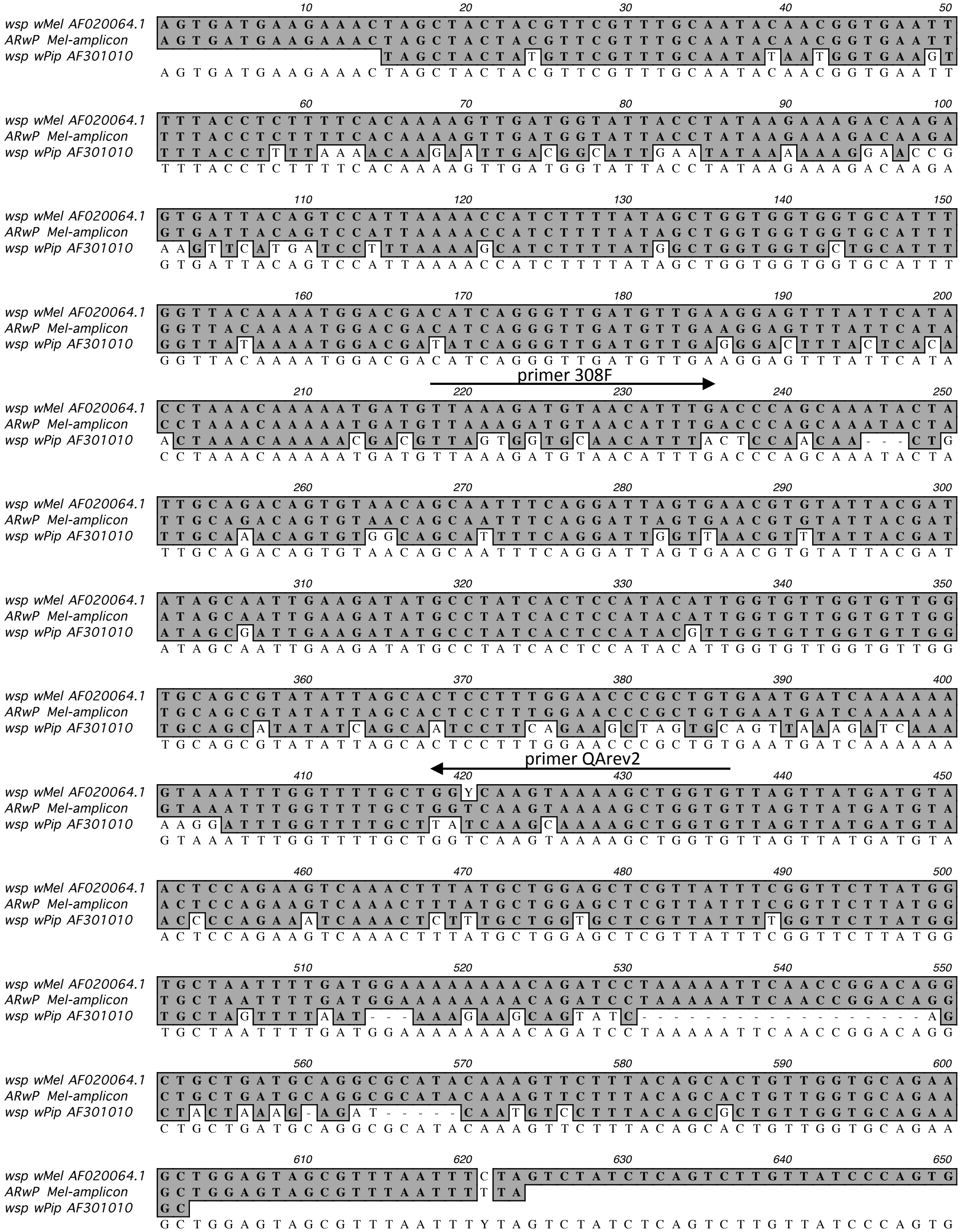
wsp wMel AF020064.1
ARwP Mel-amplicon
wsp wPip AF301010
primer 308F
primer QArev2
