# Supplementary material for: Combining Wolbachia-induced sterility and virus protection to fight Aedes albopictus-borne viruses
Source: PLoS Negl Trop Dis. 2018 Jul 18;12(7):e0006626. doi: 10.1371/journal.pntd.0006626 (PMC6066253; doi:10.1371/journal.pntd.0006626)
Supplement: S1 Table — The sequence analysis of the cloned sequences revealed a complete homology with the corresponding genes in database. (PDF) [file pntd.0006626.s003.pdf]

S1 Table. Assembling of plasmid pBS-M-P-act was performed by cloning fragments of the sequences of interest using total DNA extracts as PCR templates. The sequence analysis of the cloned sequences from the reared species listed below revealed a complete homology with the corresponding genes in database.

Gene fragment	Donor species used for cloning	Accession number of the corresponding gene in database	Percentage of homology determined by sequencing of the gene fragment
Actin	*Aedes albopictus*	DQ657949	100 %
*w*Mel-*wsp*	*Drosophila melanogaster*	AF020064	100 %
*w*Pip-*wsp*	*Culex pipiens pipiens*	AF301010	100 %
